# NOD2 receptor is crucial for protecting against the digestive form of Chagas disease

**DOI:** 10.1371/journal.pntd.0008667

**Published:** 2020-09-28

**Authors:** Nathalie de Sena Pereira, Tamyres Bernadete Dantas Queiroga, Denis Dantas da Silva, Manuela Sales Lima Nascimento, Cléber Mesquita de Andrade, Janeusa Trindade de Souto, Mayra Fernanda Ricci, Rosa Maria Esteves Arantes, Dario Simões Zamboni, Egler Chiari, Antônia Cláudia Jácome da Câmara, Lúcia Maria da Cunha Galvão, Paulo Marcos Matta Guedes

**Affiliations:** 1 Department of Parasitology, Federal University of Minas Gerais, Belo Horizonte, Brazil; 2 Potiguar University, Natal, Brazil; 3 Department of Microbiology and Parasitology, Federal University of Rio Grande do Norte, Natal, Brazil; 4 Department of Biomedical Sciences, University of Rio Grande do Norte State, Mossoró, Brazil; 5 Department of Pathology, Federal University of Minas Gerais, Belo Horizonte, Brazil; 6 Department of Cellular and Molecular Biology and Pathogenic Bioagents, Medical School of Ribeirão Preto, University of São Paulo, Ribeirão Preto, Brazil; 7 Department of Clinical and Toxicological Analyses, Federal University of Rio Grande do Norte, Natal, Brazil; University of Texas at El Paso, UNITED STATES

## Abstract

Digestive and cardiodigestive forms of Chagas’ disease are observed in 2% to 27% of the patients, depending on their geographic location, *Trypanosoma cruzi* strain and immunopathological responses. The aim of this work was to evaluate the role of NOD2 innate immune receptor in the pathogenesis of the digestive system in Chagas’ disease. Patients with digestive form of the disease showed lower mRNA expression of NOD2, higher expression of RIP2 and α-defensin 6, compared to indeterminate form, detected by Real-time PCR in peripheral blood mononuclear cells. In addition, there was a negative correlation between the expression of NOD2 and the degree of dilation of the esophagus, sigmoid and rectum in those patients. The infection of NOD2^-/-^ mice with *T*. *cruzi* strain isolated from the digestive patient induced a decrease in intestinal motility. Histopathological analysis of the colon and jejunum of NOD2^-/-^ and wild type C57BL/6 animals revealed discrete inflammatory foci during the acute phase of infection. Interestingly, during the chronic phase of the infection there was inflammation and hypertrophy of the longitudinal and circular muscular layer more pronounced in the colon and jejunum from NOD2^-/-^ animals, when compared to wild type C57BL/6 mice. Together, our results suggest that NOD2 plays a protective role against the development of digestive form of Chagas’ disease.

## Author summary

Chagas disease is caused by the protozoan *Trypanosoma cruzi*, during the chronic phase of infection 2–27% of patients develop digestive form of the disease (megaesophagus and megacolon) that contributes to patient morbidity and mortality, generating costs for public health services, and especially affecting significantly the life quality of the patients. Although is known that many factors inherent of the parasite (tropism, genetics, virulence and antigenicity), host (age, gender, nutritional status, genetics and immune response) and geographical distribution may influence the development of the different clinical forms of Chagas disease, the exact mechanism that leads to megacolon and megaesophagus development are unknown. Here we showed that patients with digestive form of Chagas’ disease do not express the innate immune receptor NOD2. By isolating a parasite from a digestive patient and infecting NOD2-deficient mice we observed a reduced intestinal motility, chronic development of colon and jejunum wall thickness associated with increased inflammatory mediators in the organ, when compared to wild type animals. Our results indicate that the NOD2 receptor protects against the development of the digestive form of Chagas disease and could be used as a biomarker for the development of gastrointestinal changes during *T*. *cruzi* infection in patients.

## Introduction

Chagas disease is caused by *Trypanosoma cruzi* and affects 6–7 million people mainly in Latin America [1]. Patients chronically infected can remain asymptomatic (39–78%), characterizing the indeterminate chronic form of the disease, or develop cardiac (17–50%), digestive (3–27%) or cardiodigestive (2–11%) forms. In the digestive form of the disease, there is mainly involvement of the esophagus and colon, due lesions in the intramural enteric nervous system, tissue parasitism and chronic inflammation leading to the appearance of megaesophagus and megacolon, respectively [2–13].

Pathological alterations of megacolon and megaesophagus include hypertrophy of the circular muscular layer, focal inflammatory reactions in the vicinity of the myenteric plexus and in the muscular layer, and fibrosis of the myenteric plexus [14]. Megaesophagus and megacolon are generated when the denervation of myenteric plexus exceeds a critical level of, at least, 85% and 50%, respectively [15–17]. It is suggested that smooth muscles are sensitive to different stimuli, and the absence of moderating action of the myenteric plexus would make the smooth muscle hyperactive, contracting disorderly what could lead also to wall hypertrophy altering the motility of several segments of the digestive tract, in particular the esophagus and colon [18–20]. In mice models proinflammatory cytokines such as IL-12, IFN-γ and TNF-α are responsible for induction of inducible nitric oxide synthase (iNOS) expression and nitric oxide (NO) production by macrophages, which contribute to the neurotoxic effects of the disease [21–25]. Denervation occurs in an irregular way and in variable intensity, due to factors related to the parasite and to the host, which have not yet been completely elucidated [26, 27].

Our group demonstrated increased of TLR8 and IFN-β expression in peripheral blood mononuclear cells of patients with digestive and cardiodigestive form of the disease [28]. TLRs activation leads NF-κB activation and production of inflammatory mediators, such as pro-IL1β, pro-IL-18, IL-6, IL-12 and TNF-α [29]. This inflammatory process contributes for the mechanism of myenteric neuronal reduction leading to megacolon and megaesophagus formation [30]. Crohn’s disease and ulcerative colitis present an increase in the expression of TLR8 and inflammatory cytokines, such as type I interferon, IL-1β, IL-6, IL-12 and TNF-α [31–34]. Moreover, several studies have demonstrated that the susceptibility to inflammatory bowel diseases is related to NOD2 deficiency [35–39], what led us to investigate the role of NOD2 in the development of morphological inflammatory-induced alterations of the digestive system in Chagas’ disease.

The NOD2 molecule is an innate immune receptor, expressed in the cytoplasm of macrophages, dendritic cells and Paneth cells, where it maintains intestinal homeostasis by binding mainly muramyl dipeptide (MDP), present in all types of bacteria [37, 38], and inducing the production of antimicrobial peptides, such as α-defensins (5 and 6) through the RIP2 signaling. In fact, the absence of NOD2 in mice and patients leads to the presence of potentially invasive bacteria, generating more frequent lesions in digestive epithelial tract than those with normal NOD2 expression [40–43].

Although studies have demonstrated the importance of inflammation and the presence of the parasite in the esophagus and colon in the pathophysiological process of megacolon and megaesophagus generation in chronic chagasic patients [14, 44–46], the mechanism by which some patients develop the anatomopathological alterations is unclear. In this study, we have demonstrated the importance of the NOD2 receptor in protecting against the inflammatory component of digestive form of Chagas’ disease. The better understanding of the pathophysiological mechanisms involved in the genesis of megaesophagus and megacolon can lead to reduction in the morbidity and mortality associated with the digestive form of Chagas’ disease.

## Methods

### Ethics statement

All patients included in this study signed the written informed consent form. The study was performed according to human experimental guidelines of the Brazilian Ministry of Health (Resolution 466/12-CNS/MS) and the Helsinki Declaration. This study was approved by the Ethics and Research Committee of the State University of Rio Grande do Norte (UERN) protocol No. 027.2011, and a Certificate of National System of Ethics in Research (CAEE-SISNEP) with protocol number 0021.0.428.000–11.

All procedures and experimental protocols performed with animals were conducted in accordance with the directives issued by the Brazilian College of Animal Experimentation (COBEA). The animal experimental protocol was approved by the Ethics Committee on Animal Use (CEUA) of the Federal University of Rio Grande do Norte (UFRN), protocol No. 23/2015.

### Patient study population

The population consisted of 80 individuals aged 18 to 79 years old. Population was selected from 10 municipalities in the Rio Grande do Norte state in Brazil: Alexandria, Apodi, Caraúbas, Governador Dix-Sept Rosado, Ipanguaçu, Mossoró, Pendências, Rodolfo Fernandes, Serra do Mel and Severiano Melo. All the individuals were submitted to serological screening for *T*. *cruzi* infection analysis. Indirect hemagglutination (Chagatest), recombinant ELISA (Wiener Lab, Rosário, Argentina) and in house indirect immunofluorescence reaction with epimastigotes of *T*. *cruzi* strain Y [47] were performed. Samples with inconclusive results were submitted to Western blot confirmatory serology (TESAcruzi, BioMérieux, Brazil) [48]. The results were considered positive when the sample was reactive in at least two of the selected methods, according to the recommendations of the World Health Organization and the Brazilian Consensus on Chagas disease [5].

Positive patients were clinically evaluated and the clinical forms were characterized using electrocardiogram (ECG), two-dimensional transthoracic echocardiography, chest X-rays and contrasted esophagus and colon (opaque enema). Esophageal contrast radiography was performed in the right anterior oblique position using barium sulfate (Bariogel, Cristália Laboratory, Brazil). Esophagus changes were classified in four levels: I) unchanged caliber, discreet contrast retention; II) small/moderate increase of the caliber, contrast retention and tertiary waves; III) large caliber increase, hypotonia, poor motor activity and great retention of radiological contrast; IV) elongated esophagus lying over the diaphragm with great retention of radiological contrast—dolico-megaesophagus [49]. Contrasted colon radiographs were performed in the supine, ventral and right lateral position using barium sulfate solution via the rectum without prior bowel preparation or double contrast use [50]. The diameter of the rectum was measured in the right lateral decubitus position, and the sigmoid colon was measured in the dorsal decubitus position, or in the ventral decubitus position if necessary [7, 51].

Patients with electrocardiographic and/or echocardiographic alterations suggestive of chagasic cardiomyopathy were evaluated by Holter 24h. Thus, asymptomatic chagasic patients who presented electrocardiograms, chest X-rays, and normal esophageal and colon contrasts were categorized with undetermined clinical form (IND, n = 18). Patients who presented with electrocardiographic abnormalities suggestive of cardiac involvement, symptomatic or not, and or cardiomegaly at chest x-ray were classified as cardiac (CARD, n = 17). Patients with altered esophageal and/or colon imaging (DIG, n = 15) and individuals with cardiomyopathy associated with megacolon and/or megaesophagus with cardiodigestive form (CARDIG, n = 15). Fifteen healthy individuals with similar age were used as controls. Data characterizing the chagasic population are shown in Table 1.

**Table 1:** Clinical characteristics of chronic chagasic subjects from Rio Grande do Norte State included in this investigation.

	Indeterminate	Cardiac	Digestive	Cardiodigestive	Total
**Gender**					
Female	8/18 (44.4%)	6/17 (35.2%)	10/15 (67.7%)	5/15 (33.3%)	29/65 (40%)
Male	10/18 (55.5%)	11/17 (64.8%)	55/15 (33.3%)	10/15 (67.7%)	36/65 (60%)
**Age (years± SD)**	41.4± 10.7	49.7± 11.8	57.6± 8.9	65.9 ± 10.6	53.3± 10.2
**Chest Radiography**					
Cardiomegaly	0/18 (0%)	5/17 (29.4%)	0/15 (0%)	5/15 (33.4%)	10/65 (15.4%)
Cardiothoracic index (heart lateral diameter/chest lateral diameter ± SD)	0.43 ± 0.05	0.48 ± 0.05	0.50 ± 0.05	0.42± 0.03	N.A.
**Contrast Radiography**					
Megacolon	0/18	0/17	8/15 9 (53.3%)	9/15 (60%)	17/65 (26.1%)
Megaesophagus	0/18	0/17	3/15 (20%)	3/15 (20%)	6/65 (9.3%)
Megacólon + Megaesophagus	0/18	0/17	3/15 (20%)	4/15 (26.6%)	7/65 (10.7%)
Sigmoid size (cm± SD)	4.41±0.57	4.5±0.71	7.72±4.6	7.96±2.87	N.A.
Rectum size (cm± SD)	4.89±0.81	5.72±0.89	7.5±31	6.36±1.89	N.A.
**Echocardiogram**					
Left ventricular ejection fraction (% ± SD)	64.6 ± 3.42	55.8 ± 14.96	56.2 ± 13.84	65.0 ± 6.48	N.A.
Left ventricular mass index (g/m^2^ body surface ±SD)	97.6 ± 21.6	100.6 ± 19.8	126.5 ± 54.4	95.9 ±16.8	N.A.
Left ventricular diastolic diameter (mm ± SD)	49.4 ± 2.9	50.6 ± 6.8	51.0 ± 5.8	47.6 ± 3.6	N.A.
Left ventricular aneurysm	0/18 (0%)	3/17 (17.7%)	1/15 (6.7%)	4/15 (26.7%)	8/65 (12.3%)
**Electrocardiogram**					
Right Branch Block	0/18 (0%)	5/17 (29.5%)	4/15 (26.8%)	0/15 (0%)	N.A.
Left Branch Block	0/18 (0%)	1/17 (5.9%)	1/15 (6.7%)	0/15 (0%)	N.A.
Anterosuperior divisional block	0/18 (0%)	4/17 (23.6%)	3/15 (20.1%)	0/15 (0%)	N.A.
Atrioventricular Block	1/18 (5.6%)	3/17 (17.7%)	2/15 (13.4%)	1/15 (6.7%)	N.A.
Supraventricular extrasystoles	1/18 (5.6%)	1/17 (5.9%)	1/15 (6.7%)	0/15 (0%)	N.A.
Ventricular extrasystoles	0/18 (0%)	3/17 (17.7%)	4/15 (26.8%)	0/15 (0%)	N.A.
Ventricular repolarization change	1/18 (5.6%)	3/17 (17.7%)	1/15 (6.7%)	1/15 (6.7%)	N.A.
Low voltage of the QRS	0/18 (0%)	8/17 (47.2%)	1/15 (6.7%)	2/15 (13.4%)	N.A.

SD: standard deviation; N.A.: not applicable; % percentage

### Parasites and mice experimental infection

The RN25 isolate of *T*. *cruzi*, was obtained from hemoculture by prof. Antonia C.J. Câmara in 2013 from a 65-year-old patient with chronic digestive form, from Serra Negra do Norte city, Rio Grande do Norte state, Brazil [52]. This is the same area where we recruited the patients included in this study. Unfortunately, this patient died due complications during a surgery to remove part of the large intestine, in 2013, before this study started.

Specific Pathogen-Free NOD2 knockouts (NOD2^-/-^) [53] and their wild type controls C57BL/6 (WT) females with six weeks old and 20–25g of bodyweight were obtained from the Center for Special Mice Breeding at Ribeirão Preto Medical School (FMRP-USP). Mice were maintained in the Experimentation Bioterium at Health Sciences Center of the Federal University of Rio Grande do Norte, housed in the same room under controlled temperature (25 °C) and with a 12 h light/dark cycle. The animals were maintained in separated cages but in the same ventilated racks with filtered air, sterile water and food provided *ad libitum*. These animals were subjected to the same environmental bacteria.

Animals were intra-peritoneally inoculated with 1×10^3^ trypomastigotes of RN25 isolate (TcII) [52]. Mice were euthanized during the acute phase (19^th^ day after infection, the parasitemia peak) or in the chronic phase (12 months after infection).

### Parasitemia, survival and gastrointestinal motility

Parasitemia was determined by the method described by Pizzi [54] modified by Brener [55]. Five microliters of blood were collected from the tip of the tail and the count was performed under the optical microscope (400 ×), using laminula (22 × 22mm) in 50 random fields. Mortality was evaluated daily. Animal intestinal motility was analyzed 0, 15, 30, 90, 180 and 360 days after infection. Briefly, three hours after food deprivation, 0.3 mL of 10% aqueous suspension of charcoal in water was administered orally by gavage. The animals were observed at 5 min intervals until faeces with charcoal were eliminated (maximum time of observation was 450 min) [56, 57]. Ten animals from each group were used for these experiments, results were calculated based on evacuation time and expressed as mean ± standard deviation (SD).

### Quantification of inflammatory mediators by Real time PCR

RNA was extracted from peripheral blood mononuclear cells (PBMC) from patients and control group. Mice had RNA extracted from the colon in the acute and chronic phases (19^th^ day and 12 months after infection, respectively). The RNA was purified using Total RNA Isolation System kit (Promega, USA), Trizol Reagent (Invitrogen, USA) and DNAse treatment. Purified RNA was stored at -80°C. RNA concentration and quality were analyzed using Nanodrop 2000 (Thermo Scientific, USA). cDNA was synthesized from 2μg of total RNA using the High Capacity cDNA Reverse Transcription kit (Applied Biosystems, USA). The reaction cycles were 10 min at 25°C, 120min at 37°C, 5min at 85°C and infinite at 4°C. PCR reactions were performed using the SYBR Green system in a 7500 Fast Real time thermal cycler (Applied Biosystems, USA). Standard Real Time PCR conditions were as follows: 50°C (2 min) and 95°C (10 min) followed by 40 cycles of 94°C (30 s), variable annealing primer temperature (Table 2) (30 s), and 72°C (1 min). The human specific primers (NOD1, NOD2, RIP2, α-defensin 5 and α-defensin 6) and mice (TLR2, TLR4, IL-10, IL-17, T-bet, TNF-α, IFN-γ, iNOS, defensin-A) used are described in Table 2. The expression mRNA levels were determined using the mean Ct values from triplicate measurements to calculate the relative expression levels of the target genes in the chagasic patients or infected mice compared to those in the healthy subjects or uninfected mice, respectively, and were normalized to the housekeeping gene β-actin (human) and GAPDH (mouse) using the 2^–ΔΔCt^ formula. The qPCR experimental protocol was described in previous work [28].

**Table 2:** Sequences of the primers used for RT-PCR reactions.

Targets	Sense and antisense sequences	Primer annealing temperature
Hu- β-actin^1^	TGACTCAGGATTTAAAAACTGGAA CACATTGTGAACTTTGGG	56.5°C
NOD1^1^	GTGGACAACTTGCTGAAGAATGAC CTGTACCAGGTCCAGAATTTTGC	60.2°C
NOD2^1^	GCCACGGTGAAAGCGAAT GGAAGCGAGACTGAGCAGACA	59.6
RIP2^1^	TGCCACCTGAAAACT-ATGAACCT ACACTTCCCATGTGATAACTGCAT	58.4
α-Defensin 5^1^	GCCATCCTTGCTGCCATT GCTTCTGGGTTGTAGCCTCATC	59.6
α-Defensin 6^1^	CCACTCCAAGCTGAGGAT CTCTGCAAAGGAGACGGC	58.4
GAPDH^2^	TGCAGTGGCAAAGTGGAGAT CGTGAGTGGAGTCATACTGGAA	58.8
TLR2^2^	CGAGTGGTGCAAGTACG GGTAGGTCTTGGTGTTCATTATC	57.7
TLR4^2^	CCTCTGCCTTCACTACAGAGACTTT GGATCATTTCCGATAAGGCT	60.9
IL-10^2^	TGGACAACATACTGCTAACC GGATCATTTCCGATAAGGCT	55.7
IL-17^2^	AGTTTGGGACCCCTTTACAC TCTCATCCAGCAAGAGATCC	57.8
T-bet^2^	CCCACAAGCCATTACAGGATG TATAAGCGGTTCCCTGGCATG	59.9
TNF-α^2^	TGTGCTCAGAGCTTTCAACAA CTTGATGGTGGTGCATGAGA	56.9
IFN-γ^2^	GCATCTTGGCTTTGCAGCT CCTTTTTCGCCTTGCTGTTG	57.6
iNOS^2^	CGAAACGCTTCACTTCCAA TGAGCCTATATTGCTGTGGCT	56.7
Defensin A^2^	GGTGATCAGCATACCCCAGCATCAGT AAGAGAAAACTACTGAGGAGCAGC	57.5

^1^Human primer,

^2^ Mouse primer.

### Histopathological analysis

Ileum, jejunum and colon of the animals were sectioned longitudinally for content removal, placed in Bouin’s solution 2% acetic acid for 10 minutes for pre-fixation as described by Arantes & Nogueira [58]. The pieces were rolled and fixed in 10% formol diluted in PBS for 24 hours. The rolls were routinely processed and paraffin embedded. Sections with 4 μm were obtained by microtomy and mounted in slides for Hematoxylin and Eosin (HE) staining. The slides were analyzed and images photographed by using the optical microscope (Olympus BX 51) equipped with Image-Pro Express 4.0 software (Media Cybernetics, MO, USA). Images were analyzed in the KS300 software (Zeiss, Jena, Germany) to determine the thickness of the jejunum and the colon muscular layer (μm). We obtained the average of three measurements for each one of 15 images obtained with the 20× objective from each animal. Semi-quantitative analysis was performed to determine inflammation and parasitism in colon tissue of C57BL/6 and NOD2^-/-^ in the acute (19 days after infection) and chronic (12 months after infection) phases. Total number of inflammatory foci (characterized by the presence of at least 10 inflammatory cells) and amastigote nests in the intestinal muscle layer were quantified in 30 fields (200× magnification) from each animal with an optical microscope (Olympus BX51). Six animals for each group were used for histopathology and the PCR experiments.

### Statistical analysis

All analyzes were performed on PRISM 9.0 software (GraphPad, San Diego, CA, USA). The Agostino-Pearson and Kolmogorov-Smirnov tests were used to verify the distribution of the data. ANOVA and Tukey-Kramer tests were performed to verify the difference between the groups with normal distribution, in the samples with non-parametric distribution the Kruskall-Wallis and Dunns test were used. The correlations of parameters analyzed in the patients were determined by the Spearman test. Differences were considered significant when p <0.05.

## Results

### Digestive form of Chagas disease is correlated to deficient NOD2 expression in patients

**Fig 1:**
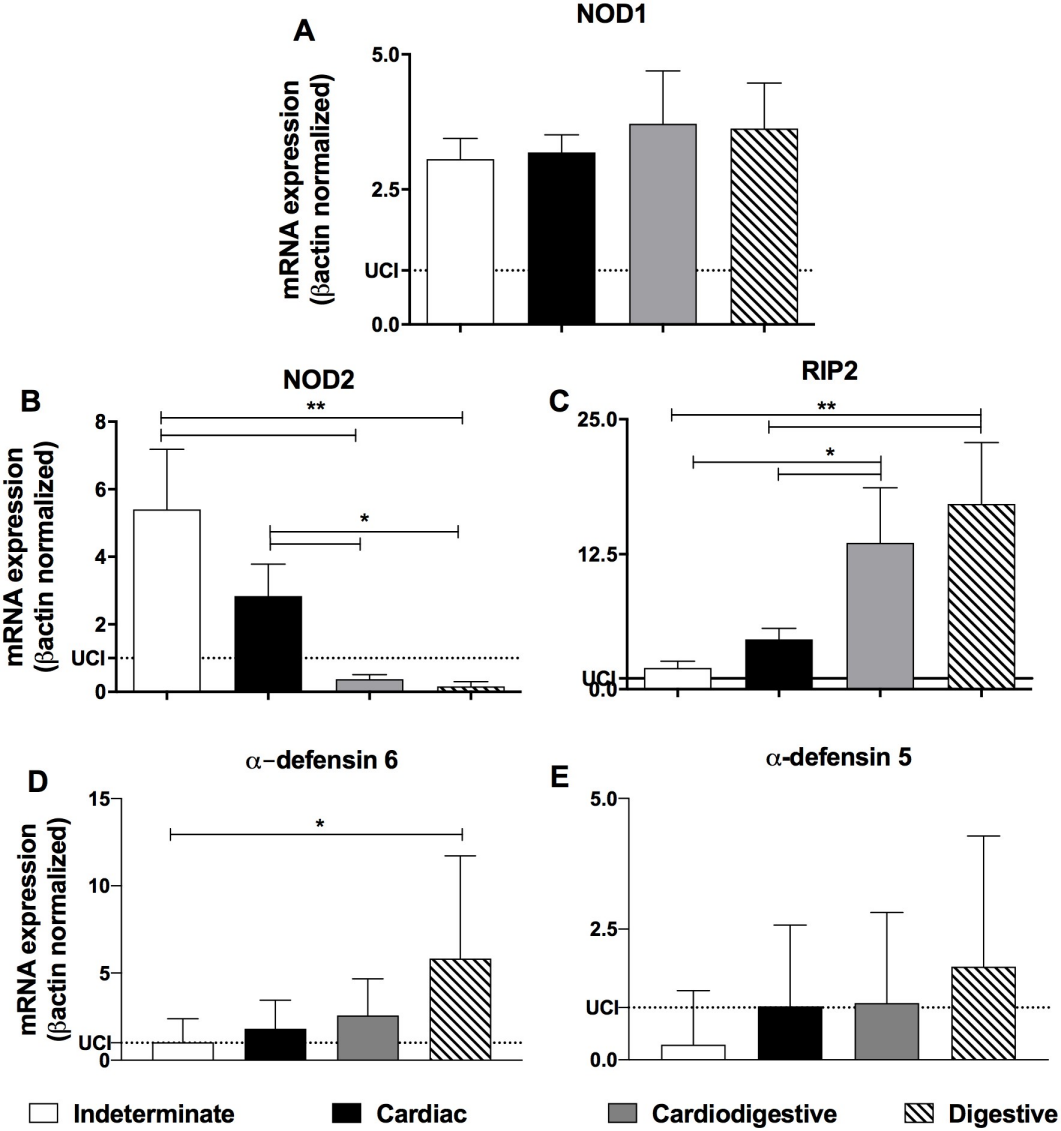
Patients with digestive form of Chagas disease showed low NOD2 and high RIP2 and α-defensin 6 expression. The mRNA expression levels of NOD1 (A), NOD2 (B), RIP2 (C), α-defensin 6 (D) and α-defensin 5 (E) were determined by real-time PCR in peripheral blood mononuclear cells of patients with the indeterminate (n = 18), cardiac (n = 17), cardiodigestive (n = 15) and digestive (n = 15) clinical forms of Chagas disease. The expression levels were normalized to the expression level of β-actin. The results are expressed as the means ± standard errors. *p < 0.05; **p < 0.01. Dotted lines represent uninfected control individuals (UCI, n = 15).

Chagasic patients (n = 65) were classified as indeterminate (n = 18), cardiac (n = 17), digestive (n = 15) and cardiodigestive (n = 15) clinical forms according to electrocardiogram (ECG), two-dimensional transthoracic echocardiography, chest X-rays and contrasted esophagus and colon (opaque enema) analysis (Table 1). Aiming to analyze the expression level of the intracellular receptors NOD1 e NOD2, its adapter molecule and products induced, a real time PCR was performed in the PBMC samples from all groups of patients. Chagasic patients with the indeterminate, cardiac, digestive and cardiodigestive clinical forms of the disease had similar NOD1 mRNA expression (Fig 1A). On the other hand, patients with the digestive and cardiodigestive forms presented absence or drastic reduced expression of NOD2 mRNA, when compared to those with indeterminate and cardiac forms (Fig 1B). Curiously, those same patients expressed higher levels of RIP2 mRNA compared to indeterminate and cardiac Chagas’ disease patients (Fig 1C). Furthermore, mRNA expression of α-defensin 6 is higher in digestive than indeterminate patients, which showed similar levels than cardiac and cardiodigestive patients (Fig 1D). All group analyzed had similar expression of α-defensin 5 transcripts (Fig 1E).

**Fig 2:**
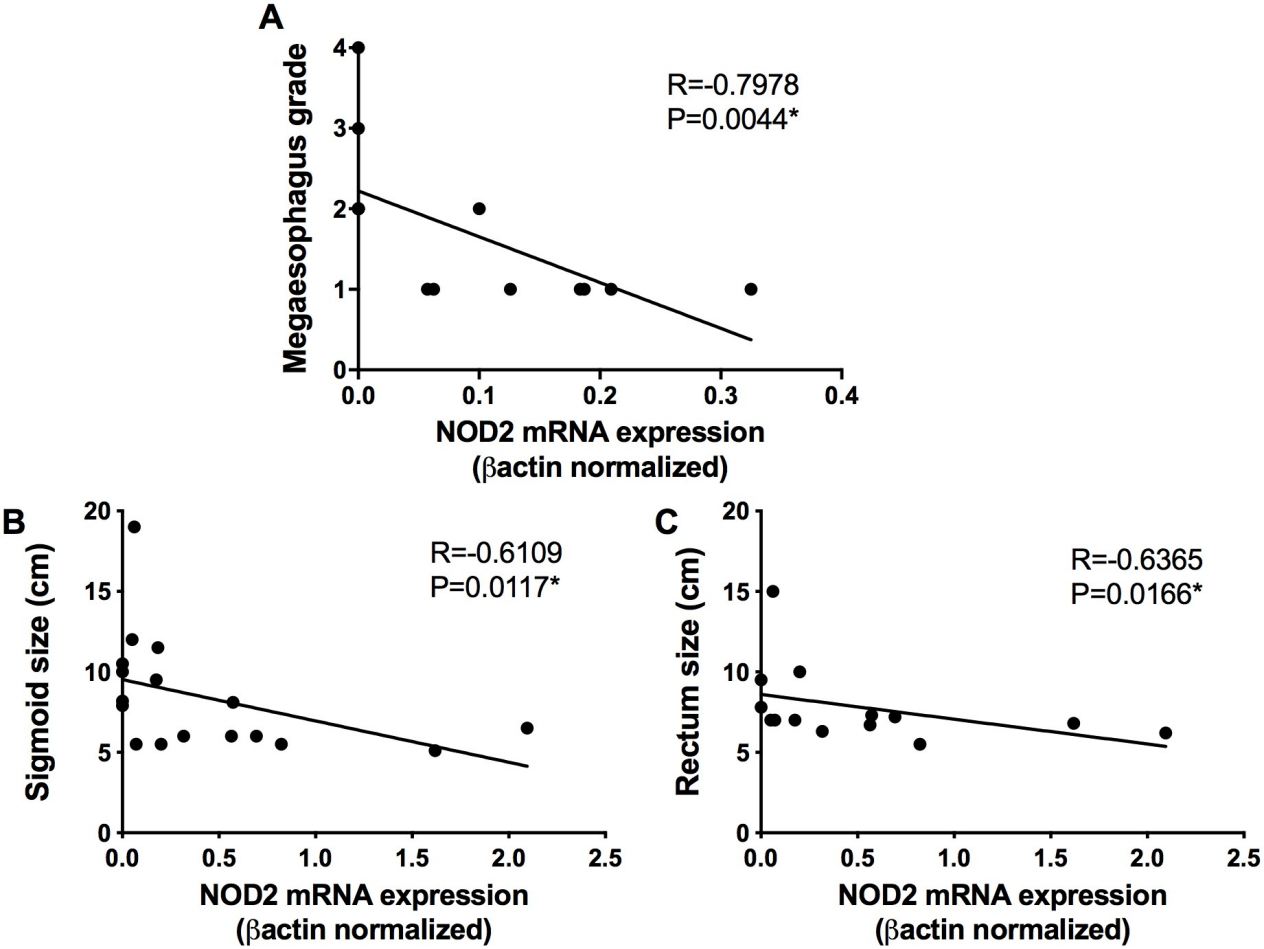
NOD2 expression is negatively correlated with the dilation degree of the esophagus, sigmoid and rectum. The mRNA expression levels of NOD2 were determined by real-time PCR in peripheral blood mononuclear cells of patients with the digestive and cardiodigestive clinical forms of Chagas`disease and correlated with megaesophagus grade (n = 13) **(A),** sigmoid size (n = 17) **(B)** and rectum size (n = 17) **(C).** The mRNA expression levels were normalized to the expression level of β-actin. Spearman test was used.

Digestive and cardiodigestive patients had the esophageal dilation, sigmoid dimension and rectum size measured by contrasted esophagus and colon (opaque enema). A negative correlation was observed between the expression of NOD2 mRNA and the degree of esophageal dilation (R = -0.7978; p = 0.0044) (Fig 2A), the sigmoid colon dimension (R = -0.6109; p = 0.0177) (Fig 2B) and rectum size (R = -0.6365; p = 0.0166) (Fig 2C) in those patients. Together, the data indicate that the deficiency in NOD2 expression is found in chagasic patients with dilation of the esophagus, colon and rectum.

### NOD2^-/-^ mice has reduced intestinal motility and hypertrophy of the jejunum and colon during chronic phase of *T*. *cruzi* infection

**Fig 3:**
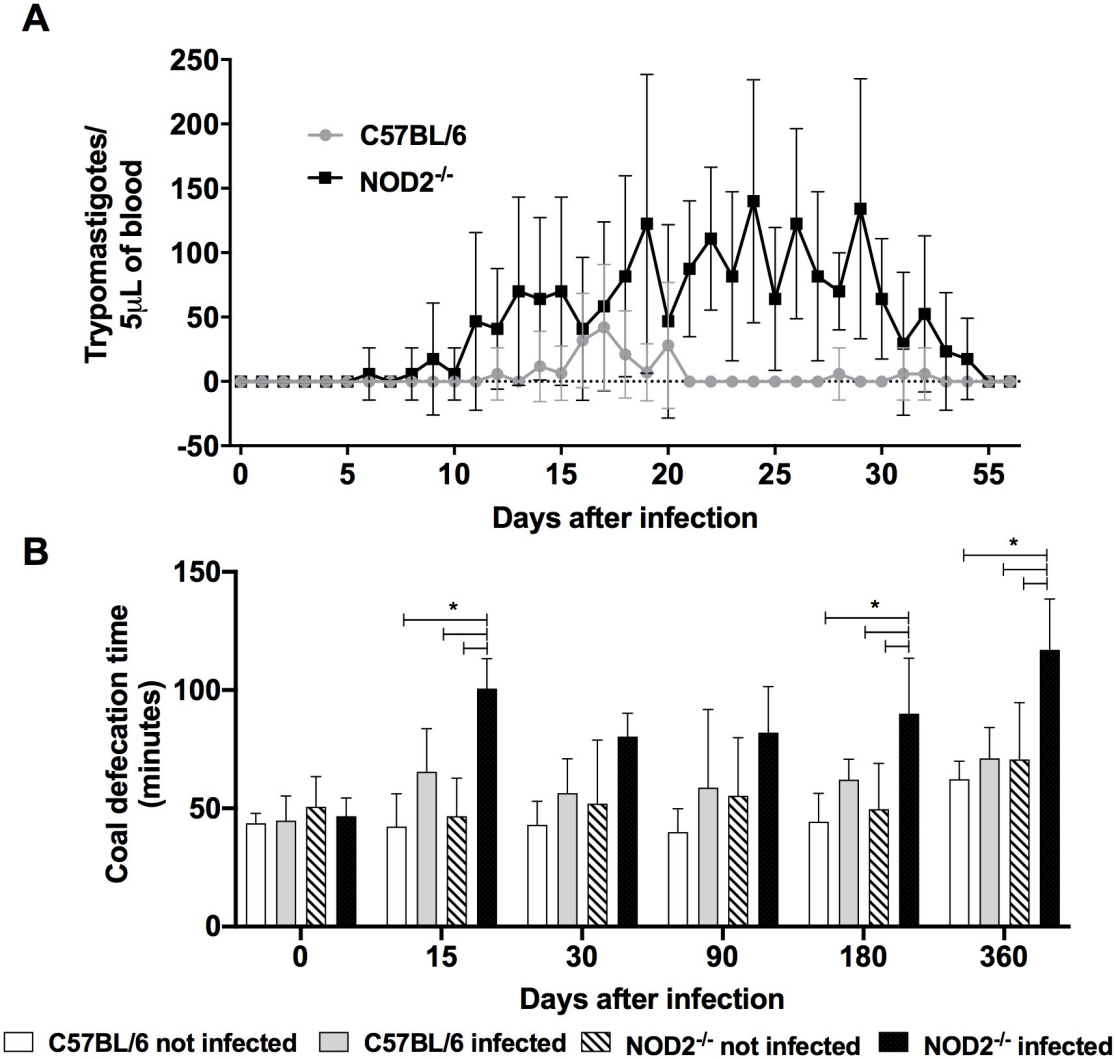
NOD2^-/-^ mice infected with *Trypanosoma cruzi* showed reduced intestinal motility in acute and chronic phases of infection. Parasitemia (A) and survival (B) of female, C57BL/6 and NOD2^-/-^ mice infected with the intraperitoneal route with 10^3^ trypomastigote blood forms of RN25 isolate (Tc-II) of *Trypanosoma cruzi*. Gastrointestinal motility was evaluated by the mean time of elimination of activated coal in infected and non-infected C57BL/6 and NOD2^-/-^ mice. The data are representative of 2 independent experiments with n = 10; * p <0.05.

To analyze the role of NOD2 receptor in the gastrointestinal lesion development during *T*. *cruzi* infection, we took advantage from the experimental model in transgenic mice. The RN25 *T*. *cruzi* isolated from a patient with a digestive form of Chagas Disease in a previously work [52] was inoculated in NOD2-deficient mice in an attempt to reproduce gastrointestinal changes observed in Chagas`patients with digestive clinical form. C57BL/6 mice were used as wild type (WT) control. Parasitemia, survival and gastrointestinal motility were evaluated. NOD2^-/-^ mice presented higher parasitemia than C57BL/6 animals between the 21^st^ and 30^th^ day after infection (Fig 3A). Patent parasitemia was observed up to 40 and 50 days in the C57BL/6 and NOD2^-/-^ infected mice, respectively (Fig 3A). However, 100% of survival was observed in both NOD2^-/-^ and C57BL/6 mice infected by *T*. *cruzi*. NOD2^-/-^ mice showed decreased intestinal motility at 15, 180 and 360 days after infection, when compared to C57BL/6 animals (Fig 3B), indicating intestinal alterations.

**Fig 4:**
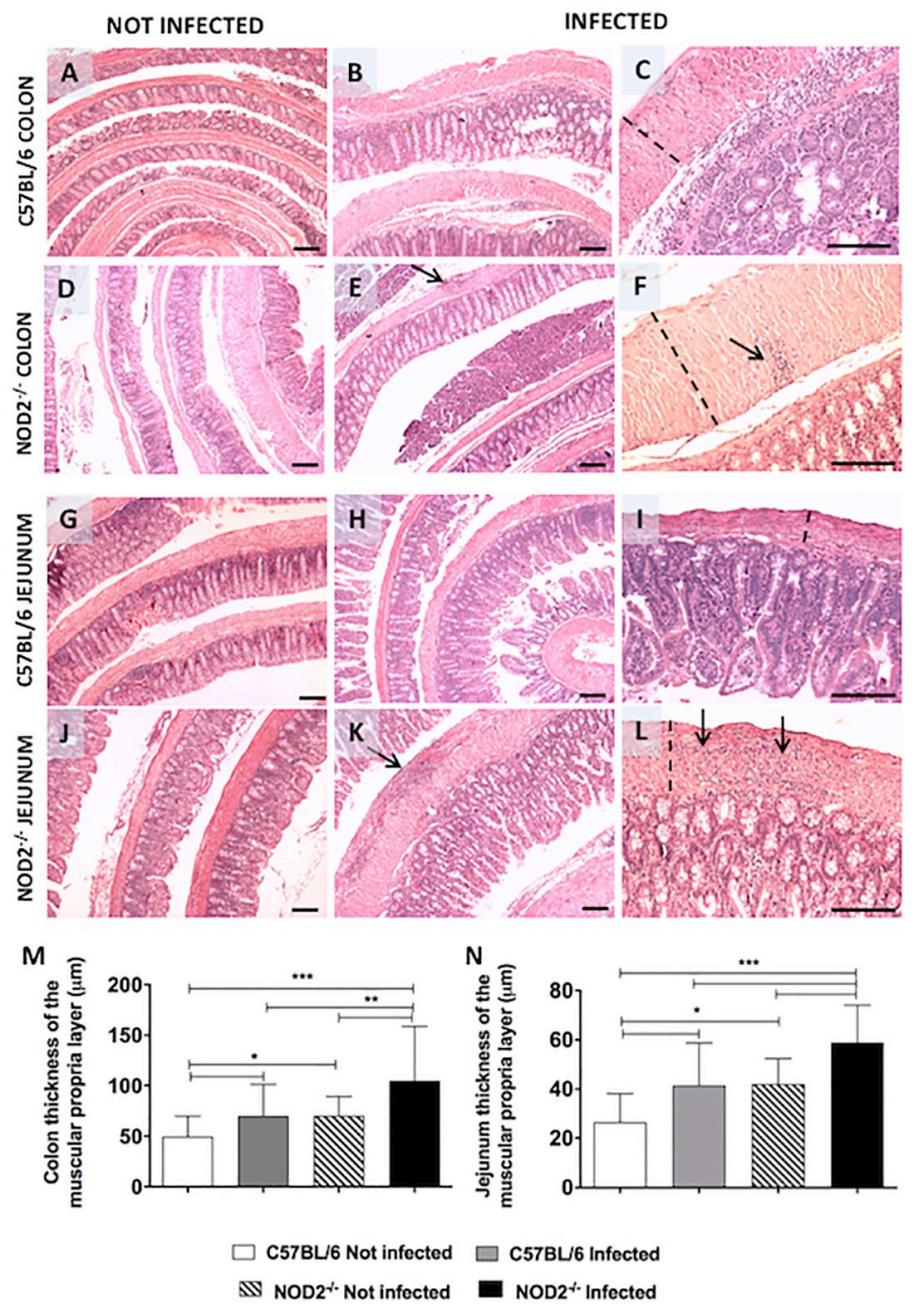
NOD2^-/-^ animals show moderate inflammation and increased thickness of the longitudinal and circular muscular layer in the colon and jejunum during the chronic phase of the infection. Histopathology of colon (A, B, C, D, E, F) and jejunum (G, H, I, J, K, L) of C57BL/6 (A, B, C, G, H, I) and NOD2^-/-^ (D, E, F, J, K, L) female mice not infected and intraperitoneally infected with 10^3^ blood trypomastigotes forms of RN 25 (Tc-II) strain of *Trypanosoma cruzi* are pictured in 200× (left and middle column) and 400× (right column) magnifications. Quantification of the colon (M) and jejunal wall thickness (N) were performed in six animals of each group euthanized after 12 months. Scale bar = 50 μm. Arrows = inflammatory foci. Dotted lines indicate muscle wall thickness. The data are representative of two independent experiments. The bars graphs are plotted as mean ± SD *p < 0.05; **p < 0.01; ***p < 0.001.

**Fig 5:**
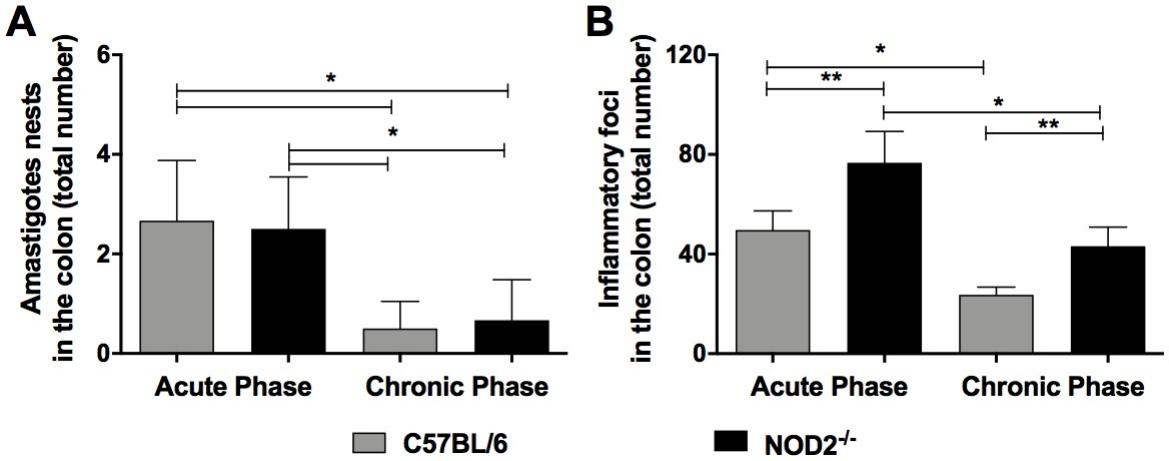
NOD2^-/-^ animals showed similar parasitism and increased inflammation in the colon during the acute and chronic phases. Quantification of amastigote nests (A) and inflammatory foci (B) were performed by count 30 fields (200× magnification in optical microscope) from H&E slides of the colon of C57BL/6 and NOD2^-/-^ mice infected. Acute (19 days) and chronic (12 months) phases are shown. n = 6 for each group. Data are representative of two independent experiments and are expressed as mean ± SD. *p < 0.05; **p < 0.01.

In attempt to evaluate the tissue parasitism, inflammation and hypertrophy of the gastrointestinal tract, histopathological analysis was performed in WT and NOD2^-/-^ in the acute (19 days) and chronic phases (12 months) of infection (Figs 4A–4N, 5A and 5B). The infection in the NOD2^-/-^ animals caused intense focal inflammation in the colon during the chronic phase (Fig 4E, 4F and 4M), when compared to C57BL/6 mice (Fig 4B, 4C and 4M). Jejunum was the most affected region of the intestine, with intense inflammation in the NOD2^-/-^ mice (Fig 4K, 4L and 4N) compared to C57BL/6 mice (Fig 4H, 4I and 4N). The inflammatory infiltrate presents a predominance of mononuclear cells, reaching the entire thickness of the muscular layers, sometimes forming cellular cords between the muscular layers and covering the ganglionic elements of the enteric nervous system. The colon of the NOD2^-/-^ and C57BL/6 infected animals presented increased thickness of the muscularis propria, when compared to uninfected animals. However, NOD2^-/-^ infected mice showed a more marked increase in the colon thickness of the muscularis propria than C57BL/6 infected animals (Fig 4C, 4F and 4M). Uninfected NOD2^-/-^ mice presented a discrete increase in colonic wall thickness when compared to uninfected C57BL/6 animals (Fig 4M). The mice infection with a parasite isolated from a patient with digestive form of the disease led to an increasing in the jejunum wall thickness in both NOD2^-/-^ and C57BL/6 infected mice, when compared to uninfected controls (Fig 4N), but the jejunum wall thickness in NOD2^-/-^ infected animals was bigger when compared with C57BL/6 infected mice (Fig 4I, 4L and 4N). C57BL/6 and NOD2^-/-^ mice show similar parasitism during the acute and chronic phases of infection (Fig 5A). The parasitism was higher in acute than in chronic phase in both groups (Fig 5A). Semiquantitative analysis of the inflammatory foci in colon fragments of *T*. *cruzi*-infected mice demonstrated that NOD2 deficient animals present high inflammation in the colon during the acute and chronic phases, when compared to C57BL/6 animals (Fig 5B). These data emphasize the importance of NOD2 receptor in preventing the development of gastrointestinal tract alterations in *T*. *cruzi*-infected mice.

### Reduced intestinal motility and intestine hypertrophy of NOD2^-/-^ is correlated to enhanced inflammatory markers in the colon during acute and chronic phases

**Fig 6:**
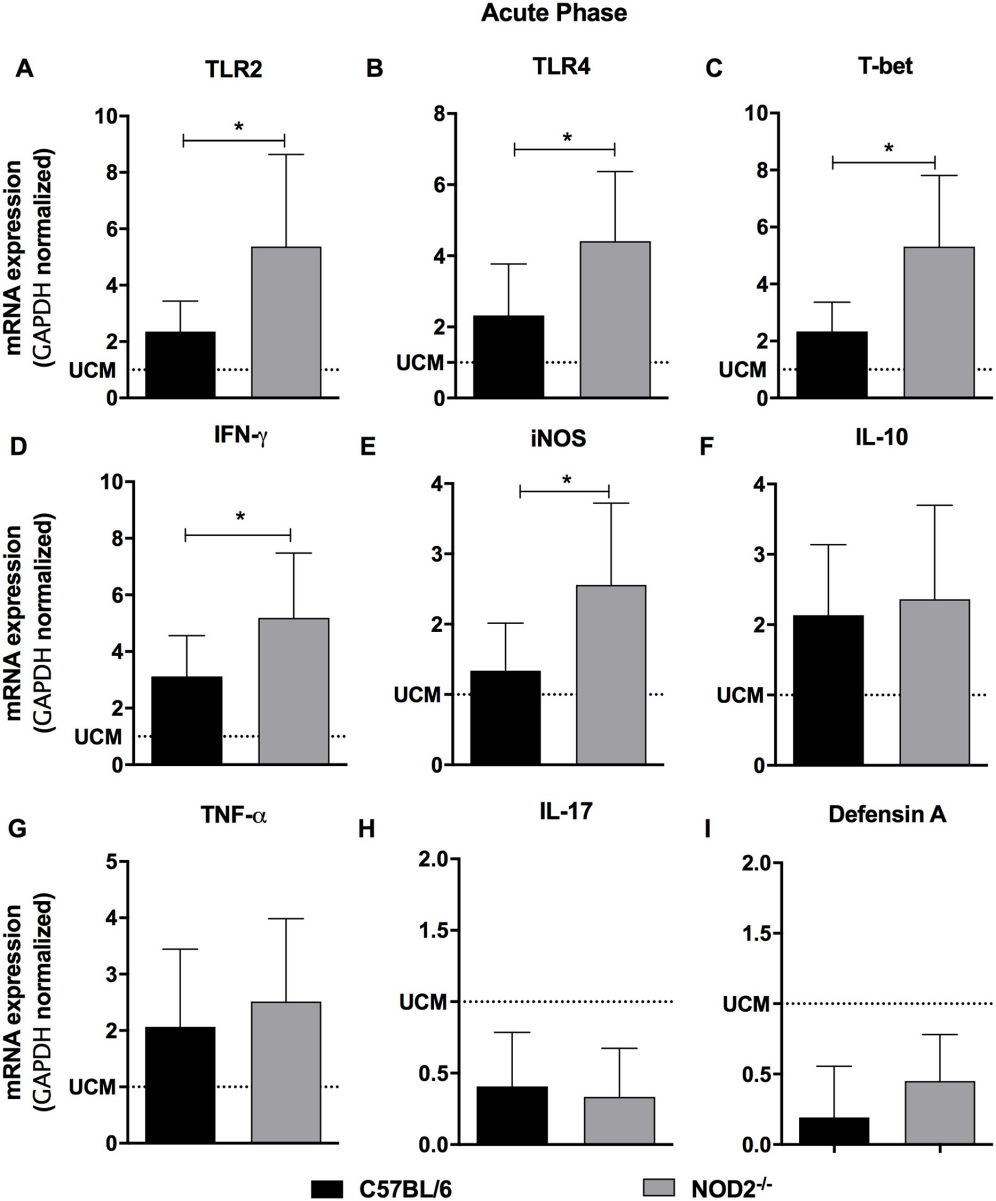
NOD2^-/-^ mice presented high expression of inflammatory markers in the colon during the acute phase of *Trypanosoma cruzi* infection. The expression of TLR2 (A), TLR4 (B), T-bet (C), IFN-γ (D), iNOS (E), IL-10 (F), TNF-α (G), IL-17 (H) and defensin-A (I) were determined by real-time PCR in the colon of C57BL/6 and NOD2^-/-^infected mice and euthanized 19 days after infection. Data are representative of two independent experiments and expressed as mean ± SD. * p <0.05. Dotted lines represent uninfected control mice (UCM). n = 6 for each group.

**Fig 7:**
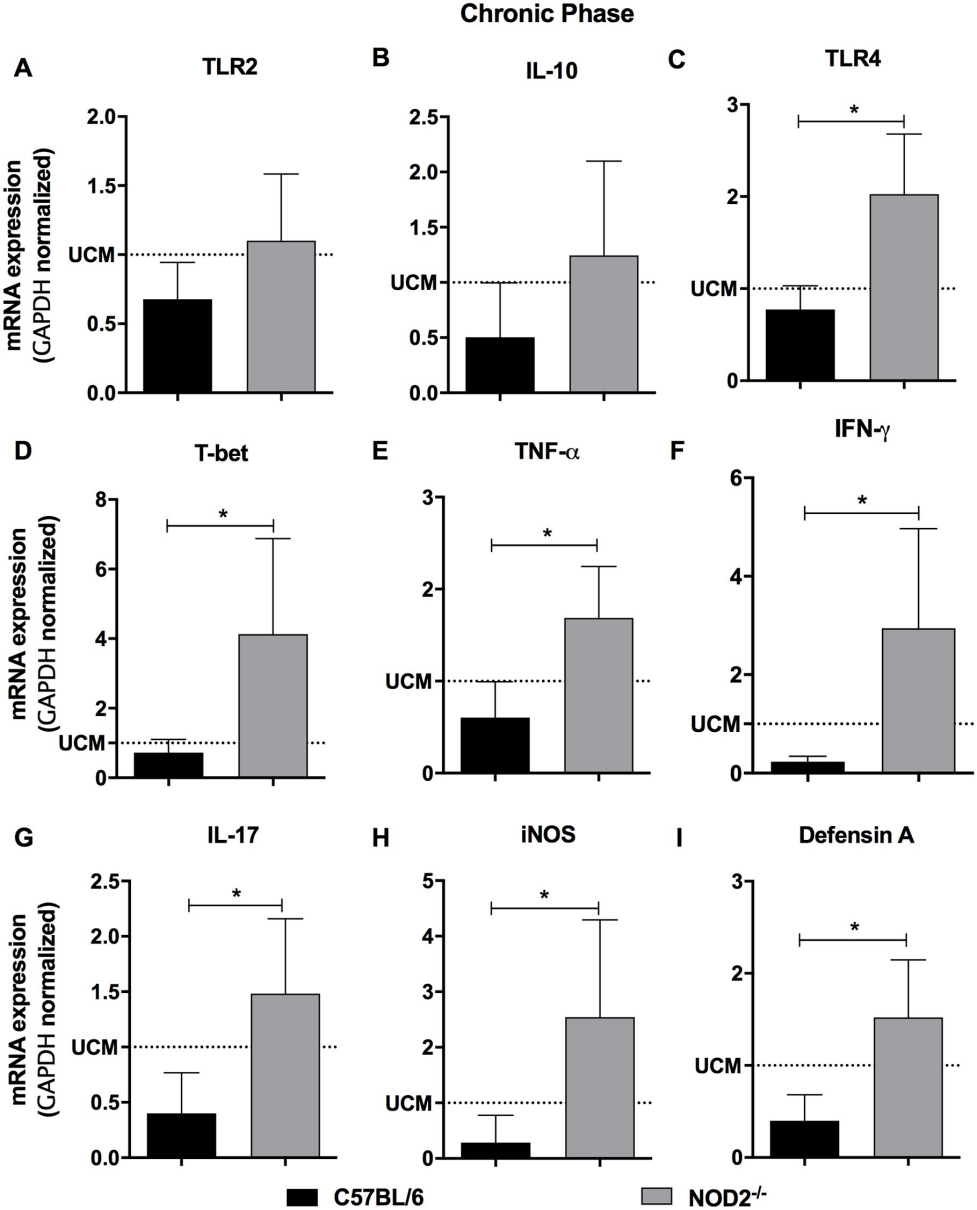
NOD2^-/-^ mice presented high expression of inflammatory markers in the colon during the chronic phase of *Trypanosoma cruzi* infection. The expression of TLR2 (A), IL-10 (B), TLR4 (C), T-bet (D), TNF- (E), IFN-γ (F), IL-17 (G), iNOS (H) and defensin-A (I) were determined by real-time PCR in the colon of C57BL/6 and NOD2^-/-^ infected mice and euthanized 12 months after infection. Data are representative of two independent experiments and expressed as mean ± SD. * p <0.05. Dotted lines represent uninfected control mice (UCM). n = 6 for each group.

The immunopathogenic mechanisms that lead to changes in the gastrointestinal tract, megacolon and megaesophagus formation depends on the presence of the parasite, inflammation and denervation. Thus, the expression of inflammatory markers that may contribute to this process was evaluated during the acute and chronic phases after *T*. *cruzi* infection. Acute phase evaluation showed increased expression of TLR-2, TLR-4, T-Bet, IFN-γ, iNOS, IL-10, TNF-α (Fig 6A–6G), and reduced IL-17 and defensin-A (Fig 6H and 6I) mRNA expression in the colon of *T*. *cruzi* infected animals, compared to uninfected mice. Moreover, NOD2^-/-^ infected mice showed higher TLR-2, TLR-4, T-Bet, IFN-γ and iNOS (Fig 6A–6E) than C57BL/6 animals. Similar levels of IL-17 and defensin-A mRNA expression was observed in NOD2^-/-^ and C57BL/6 infected animals during acute phase of infection (Fig 6H and 6I). During the chronic phase we observed similar mRNA expression of TLR2 and IL-10 (Fig 7A and 7B) in the colon of NOD2^-/-^ and C57BL/6 *T*. *cruzi*-infected mice. On the other hand, there was increased expression of TLR-4, T-Bet, TNF-α, IFN-γ, IL-17, iNOS, and defensin-A mRNA (Fig 7C–7I) in the colon of NOD2^-/-^ when compared to WT mice. The higher expression of inflammatory mediators in NOD2 deficient mice and the decrease of IL-10 possibly contributes to the increase of musculature and appearance of lesions in the colon.

## Discussion

We evaluated the role of NOD2 receptor in the lesion genesis of the gastrointestinal tract during the experimental infection by *T*. *cruzi* and its participation in the development of the digestive clinical form in patients with Chagas’ disease. The results indicate that deficiency in the expression of the NOD2 receptor is correlated with the appearance of changes in the gastrointestinal tract during experimental infection in mice and also with the development of the digestive clinical form of Chagas’ disease in patients.

We observed here that patients with digestive and cardiodigestive forms of Chagas’ disease present absence or reduced NOD2 mRNA expression. We also demonstrated a negative correlation between the NOD2 mRNA expression with the degree of esophageal dilation, sigmoid and rectum sizes. These results indicate that NOD2 molecule has a protective role against the development of gastrointestinal tract lesions in patients with Chagas’ disease. The NOD2 receptor is highly expressed in Paneth cells being responsible for the regulation of the intestinal microbiota through the production of antimicrobial components such as defensins [29, 59]. NOD2 deficient expression influences the composition of the microbiota because its absence leads to a higher number of pathogenic bacteria in the gastrointestinal tract [60] and is also involved with the development of inflammatory bowel diseases. Patients with Crohn’s disease present alteration of the intestinal microbiota, with increase of *Bacteroidetes*, *Proteobacteria* and reduction of *Firmicutes* [61]. Patients with Chagas’ disease with megaesophagus and megacolon present increased bacterial growth in the gastrointestinal tract when compared to the microbiota of healthy individuals [62, 63]. In addition, chagasic patients with the digestive form have higher number of bacteria with pathogenic potential, which is positively correlated with esophageal dilatation [63].

In the present study, increased expression of α-defensin 6 was observed in patients with the digestive form, compared to indeterminate and cardiac patients. Defensin production is stimulated after the contact of the bacteria with the digestive tract epithelium. Besides NOD2 pathway, the α-defensin production can also be induced through Toll-Like Receptors (TLR) [64–69] in Paneth Cells. In fact, we have previously demonstrated that those same patients have intact TLR family transcripts expression, being TLR8 overexpressed in digestive patients [28]. The microbiota dysbiosis observed in digestive chagasic patients [62, 63, 70] might be crucial for the elevated α-defensin levels in attempt to play its role through the formation of nanofibrils that protect intestinal mucosa from bacterial invasion [71–73]. Even in the absence or reduced expression of NOD2 in digestive and cardiodigestive patients, we showed that its adapter molecule RIP2 was overexpressed in those patients. This can be due a compensate mechanism or as consequence of NOD1 signaling activated in an attempt to induce cytokine and NO production to control the *T*. *cruzi* [74]. The increased expression of RIP2 in digestive and cardiodigestive patients can exacerbate the pathway to induce a greater cytokines production and, consequently, inflammation that contributes to the formation of the mega syndrome.

Another feature that can contribute to megacolon and megaesophagus formation is the parasite tropism to the gastrointestinal tract. Together with microbiota dysregulation and NOD2 deficiency, the presence of the parasites generates even more tissue damage and inflammation in the intestinal and esophageal epithelium, leading to denervation and contributing to organ pathology. Thus, our data suggest that NOD2 receptor plays an important role in the protection against lesions genesis of digestive tract in chagasic patients. Is important to note that, as described by others [7, 75–79], we also observed that the presence of megacolon is more frequent than the presence of megaesophagus in these chronic chagasic patients. This aspect is influenced by the anatomy of the organ, because in the esophagus, the alimentary content passes more easily influenced by gravity, whereas in the colon and rectum it presents a natural tendency of stagnation. Also, the colon and rectum present a higher number and diversity of bacteria than the esophagus [80], which could contribute to intensify inflammation, denervation and dilatation observed in the colon and rectum.

In order to confirm the importance of the NOD2 receptor in the genesis of digestive tract lesions in Chagas’ disease, the development of gastrointestinal tract lesions was evaluated in NOD2 knockout mice infected with *T*. *cruzi* obtained from a digestive patient. Decreased intestinal motility was observed in NOD2^-/-^ mice, indicating alterations in the gastrointestinal tract. The histopathological analysis of the colon demonstrated a moderate inflammatory lesion in the sub serous and muscular layer, more pronounced in NOD2^-/-^ animals than C57BL/6 during the acute phase of infection. The lesions in the colon induced by *T*. *cruzi* during experimental infection in the acute phase include parasitism, degeneration and necrosis of muscle fibers. These are followed by intramuscular fibrosis, denervation and enlargement of the colon wall in the presence of sustained focal and moderate inflammation of the muscular propria by 15 months post infection in chronic phase [30]. NOD2^-/-^ animals presented more intense focal inflammation in the colon and jejunum wall during acute and chronic phases of infection impacting in the thickness of the muscular layers and ganglionic elements of the enteric nervous system. These results indicate that infection with a parasite isolated from a patient with digestive form generates a progressive inflammation distributed in foci throughout the entire gastrointestinal tract, which altogether contribute significantly to the denervation process during the course of the disease.

The TLR2 and TLR4 activation by GPI-anchors and glycoinositolphospholipids derived from *T*. *cruzi*, respectively, leads NFκβ activation, IL-12, IFN-γ, TNF-α, IL-17 production and iNOS activation [81–83]. Besides being important to kill the parasite, when in excess, TNF-α, IFN-γ and iNOS contribute to myenteric plexus degeneration during *T*. *cruzi* experimental infection [24]. Here, we demonstrated that NOD2-deficient mice infected with *T*. *cruzi*, which have an increased colon size, produce higher levels of these inflammatory mediators *in situ* when compared to WT animals. Interestingly, NOD2 is a negative regulator of TLR2 signaling, and then NOD2 deficiency can result in the production of Th1-related cytokines in environments rich in TLRs, such as the intestine [84]. In fact, we observed that NOD2 deficient mice showed high TLR2 and inflammatory mediators expression during the acute phase of the infection. These characteristics point to the central role of NOD2 receptor in the control of intestinal homeostasis, and could explain the increase in the long-term inflammation, which is the substrate of myenteric plexus denervation and exacerbated muscle hypertrophy generated by the *T*. *cruzi* strain with gastrointestinal tract tropism used in this investigation. Most likely both parasite tropism and chronic inflammation influence gastrointestinal tract injury.

Together, our results indicate that deficiency in NOD2 expression associated to *T*. *cruzi* strain that causes persistent intestinal inflammation promotes the breakdown of homeostasis of the digestive system, making patients more susceptible to the development of the digestive form of Chagas’ disease. Thus, it is possible to hypothesize that the bacteria present in the microbiota are able to cross the mucus and IgA barriers, coming into contact with the epithelial layer of the digestive tract and inducing or potentiating the inflammation generated by the parasite in NOD2-deficient patients. The inflammation is responsible for denervation of the intestinal tract, leading to dysmotility, incoordination and gradual loss of peristalsis [14, 46]. The intestinal tract will dilate and luminal contents will initiate the compression of the viscera generating also ischemic pathologies that will amplify the process of formation of the megaesophagus and megacolon [4, 15, 20, 45, 85]. Finally, NOD2 is a protective factor against the development of digestive form of Chagas’ disease by impacting in the control of parasite-induced inflammation and microbiota homeostasis.
